# Man with Scrotal Rupture

**DOI:** 10.5811/cpcem.2017.6.34771

**Published:** 2017-10-03

**Authors:** Andrew Perechocky, Liam Mahoney, Gina Lopez

**Affiliations:** Boston Medical Center, Department of Emergency Medicine, Boston, Massachusetts

## CASE PRESENTATION

**Image 1:**
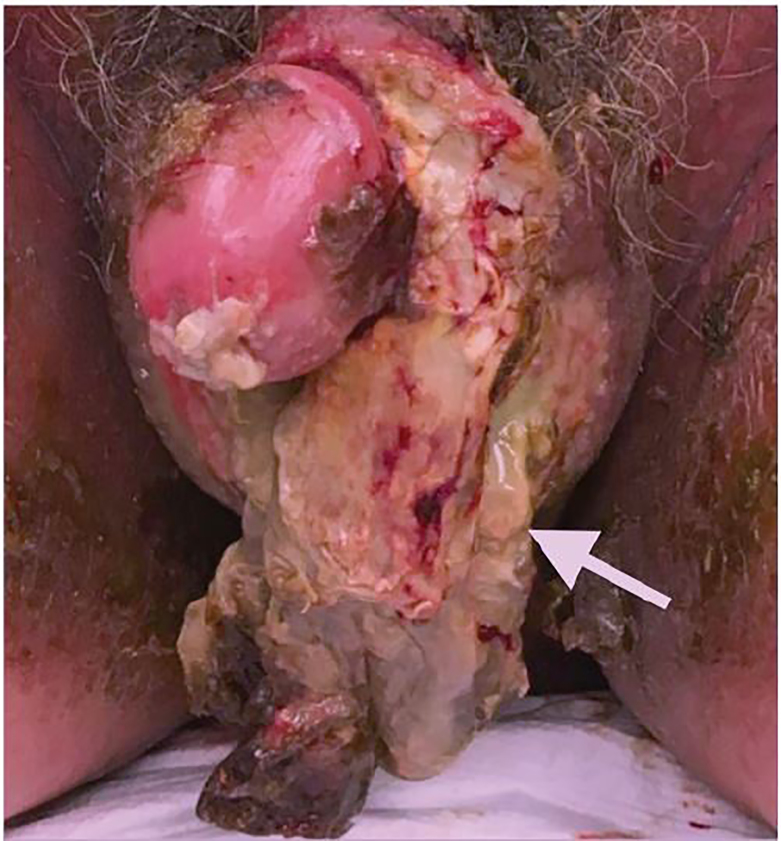
Scrotal rupture (white arrow)

**Image 2:**
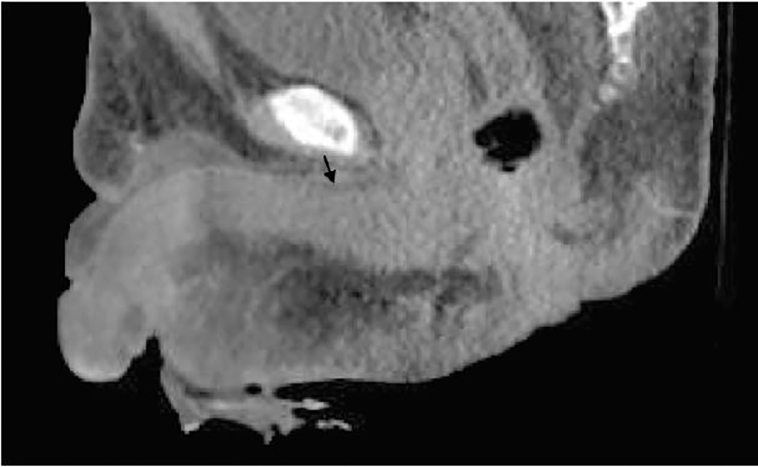
Computed tomography (sagittal view) of genitalia showing focal fluid 1.0 cm in thickness tracking along the superior aspect of the penile shaft (black arrow)

A 55-year-old male presented to the emergency department (ED) with scrotal pain and swelling. He reported that four months prior, he fell and sustained minor trauma to his scrotum. Since that time he had progressive pain and swelling of the scrotum. His past medical history was unknown, as the patient had not sought medical care in many years. That evening when he sat on the toilet he felt his scrotum “explode” and saw pus and tissue in the toilet. Physical exam was notable for a ruptured scrotum (Image 1) with frank purulence and excoriation of the ventral aspect of the penis. The patient was also noted to have pitting edema to the mid-abdomen. A computed tomography (CT) was obtained (Image 2).

## DISCUSSION

While testicular rupture is not uncommon, isolated scrotal rupture is a rare entity. It can be a consequence of blunt trauma to the genitalia,^1^ often from a sports injury or motor vehicle accident. Birth trauma in a neonate has also been reported as a cause of scrotal rupture.^2^ Scrotal rupture is a urologic emergency that requires operative repair.

This patient received antibiotics in the ED. Urology was consulted and performed a penoscrotal exploration and debridement in the operating room. Approximately 30% of the scrotum was necrotic and removed, but the testicles remained intact. The patient was admitted to the surgical intensive care unit where cardiology was consulted for assistance managing the patient’s decompensated heart failure. On day 12 the patient was discharged from the hospital with anti-hypertensive medications as well as urology and cardiology outpatient follow-up.

**CPC-EM Capsule**

What do we already know about this clinical entity?

Scrotal rupture is a rare entity typically caused by blunt trauma such as a sports injury or motor vehicle accident. It is a urologic emergency that requires operative repair.

What is the major impact of the image?

The image serves as a reference for scrotal rupture. Comparable images are not readily available to the medical community.

How might this improve emergency medicine practice?

The image raises awareness of this clinical entity for emergency physicians, urologists, surgeons, and other clinicians who care for patients with traumatic injuries.
